# Prevalence of incidentally detected pancreatic cysts on magnetic resonance imaging in an adult population in Latin America

**DOI:** 10.1590/0100-3984.2024.0103

**Published:** 2025-04-14

**Authors:** Fernando Revoredo Rego, Yuri López Zenteno, Fritz Kometter Barrios

**Affiliations:** 1 Clínica Internacional, Lima, Peru.

**Keywords:** Incidental findings, Pancreatic cyst/diagnostic imaging, Magnetic resonance imaging, Kidney diseases, cystic, Liver diseases, Cysts/diagnostic imaging, Achados incidentais, Cisto pancreático/diagnóstico por imagem, Imageamento por ressonância magnética, Doenças renais císticas, Hepatopatias, Cistos/diagnóstico por imagem

## Abstract

**Objective:**

To determine the prevalence of incidentally detected pancreatic cystic lesions (PCLs) in adult patients undergoing magnetic resonance imaging (MRI).

**Materials and Methods:**

We included radiological records of consecutive adult patients who underwent MRI at our institution during a one-year period (January to December of 2023). We collected clinical and radiological data, including the presence or absence of cysts in the liver and kidneys.

**Results:**

A total of 1,211 MRI records were included. We identified PCLs in 138 patients, corresponding to a prevalence of 11.4%. That prevalence was 9.51% in men and 12.52% in women (p = 0.112). The patients with incidental PCLs (64.57 ± 13.15) were significantly older than were those without (mean age, 64.57 ± 13.15 years vs. 51.01 ± 15.27 years; *p* < 0.001). Of the 138 patients with PCLs, 53 (38.41%) had at least one liver cyst and 83 (60.14%) had at least one kidney cyst. In 69 patients (50.0%), the radiological diagnosis of the incidental cysts was intraductal papillary mucinous neoplasm. In the univariate analysis, the presence of PCLs was associated with age, liver cysts, and kidney cysts, although it was associated with only age and kidney cysts in the multivariate analysis.

**Conclusion:**

In our study sample, the prevalence of incidentally detected PCLs was 11.4%. That prevalence increased significantly with age but did not differ by sex.

## Abstract (pt)

**Objetivo:**

Determinar a prevalência de cistos pancreáticos (CPs) detectados incidentalmente em pacientes adultos submetidos a ressonância magnética (RM).

**Materiais e Métodos:**

Foram incluídos registros radiológicos consecutivos de pacientes submetidos a RM em nossa instituição durante o período de um ano (janeiro de 2023 a dezembro de 2023). Foram coletadas informações clínicas e radiológicas e presença de cistos hepáticos e/ou renais.

**Resultados:**

Foram incluídos 1.211 registros de RM. Foram identificados CPs em 138 pacientes, constituindo uma prevalência de 11,4%. A prevalência de CPs foi de 9,51% no sexo masculino e 12,52% no sexo feminino (*p* = 0,112). A média de idade dos pacientes com CPs incidentais (64,57 ± 13,15) foi significativamente maior que a média de idade dos pacientes sem CPs (51,01 ± 15,27 anos) (*p* < 0,001). Dos 138 pacientes com CPs, 38,41% apresentavam cistos hepáticos e 60,14%, cistos renais. O diagnóstico radiológico dos cistos incidentais foi neoplasia mucinosa intraductal papilar em 69 pacientes. Idade, cistos hepáticos e cistos renais estiveram associados à presença de CPs na análise univariada, embora apenas idade e cistos renais tenham apresentado associação com presença de CPs na análise multivariada.

**Conclusão:**

A prevalência de CPs detectados incidentalmente foi de 11,4%. Esta prevalência aumentou significantemente com a idade, mas sem diferença em relação ao sexo.

## INTRODUCTION

Pancreatic cystic lesions (PCLs) are being detected with increasing frequency due to the widespread use of modern cross-sectional imaging studies and improvements in imaging technology^(1, 2, 3)^. The prevalence of PCLs varies according to the type of imaging study used^(2)^. Whereas abdominal ultrasound detects PCLs in 0.21% of patients^(4)^, computed tomography detects it in 2.6–5.0%^(5, 6)^, magnetic resonance imaging (MRI) detects it in 2.4–49.0%^(3,7)^, and endoscopic ultrasound detects it in 21.5%^(8)^. A finding of PCLs generates concerns for patients and health care providers related to potential risk of a life-threatening malignancy and is increasingly a motive for referral to specialized centers and a major cause of resource utilization^(9)^.

The PCL spectrum encompasses a broad variety of lesions^(10)^. This heterogeneous group includes the following^(1)^: epithelial neoplasms, nonepithelial neoplasms (lymphangioma, cystic pancreatic hamartoma, and mesothelial cyst), and lesions resembling pancreatic neoplasms (pseudocyst, enteric duplication cysts, hydatid cysts, and retention cysts).

The epithelial neoplasms include intraductal papillary mucinous neoplasms (IPMNs), mucinous cystic neoplasms, serous cystic neoplasms (SCNs), and some rare cystic neoplasms, such as solid pseudopapillary neoplasms, pancreatic ductal adenocarcinomas with cystic degeneration, and cystic pancreatic neuroendocrine tumors^(1, 2)^. Together, these cystic epithelial neoplasms represent around 90% of all PCLs, with IPMNs being the most common^(2)^.

There have been several MRI studies on the prevalence of PCL^(11,12)^, only one of which was carried out in South America^(12)^. In that study, conducted in Brazil, the reported prevalence of incidental PCLs was 9.3%.

The aim of this study was to determine the prevalence of incidentally detected PCLs in adult patients without known pancreatic disease who underwent MRI. We also analyzed the association between PCLs and cysts in other abdominal organs.

## MATERIALS AND METHODS

This was a retrospective observational study, in which we included the radiological records of consecutive patients who underwent MRI at our institution during a one-year period (January to December of 2023). This study was approved by the institutional review board of the institution. All procedures were carried out in accordance with the Strengthening the Reporting of Observational Studies in Epidemiology guidelines^(13^ and in compliance with the ethical principles for medical research involving human subjects established in the Helsinki Declaration^(14)^. Because of the retrospective nature of the study, the requirements for informed consent and ethics committee approval were waived.

**Figure 1:**
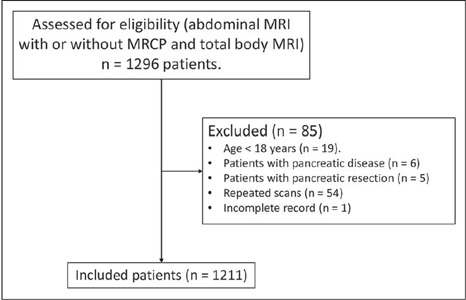
Cohort flow chart. (MRCP, magnetic resonance cholangiopancreatography).

All MRI scans were retrospectively reviewed by a specialized radiologist using dedicated software (Centricity Zero Footprint Universal Viewer; GE Healthcare, Chicago, IL, USA). The scans were reviewed in chronological order (i.e., if a patient underwent more than one MRI scan during the study period, only the first examination was included). After the MRI reports had been reviewed, electronic medical records were analyzed. All individuals were adults (≥ 18 years of age). Patients with known or suspected pancreatic disease were excluded, as were those who were under surveillance, those who had undergone pancreatic resection of any type (pancreatoduodenectomy, distal pancreatectomy, or total pancreatectomy), and those for whom the records were incomplete (Figure 1).

The following data were collected for each patient: age; sex; the presence of PCL; size, defined as the largest diameter of the cyst on either an axial or coronal T2-weighted image^(15)^; location (pancreatic head, neck, body, or tail, or multifocal); morphology; communication with pancreatic ductal system (necessary for the diagnosis of an IPMN); presence of worrisome features or high-risk stigmata; and presence of liver or kidney cysts.

High-risk stigmata and worrisome features were defined according to the international evidence-based Kyoto guidelines for the management of IPMNs^(16)^.

### MRI protocol

The MRI examination was performed in a 3.0-T scanner (Magnetom Skyra; Siemens Healthcare, Erlangen, Germany). The protocol for MRI of the upper abdomen was as follows: T2-weighted fast spin-echo sequences in the axial, sagittal, and coronal planes; axial T1-weighted fluid attenuated inversion recovery sequence with fat suppression; axial in-phase and out-of-phase T1-weighted gradient echo sequences; diffusion-weighted sequence; and apparent diffusion coefficient mapping. After administration of the contrast agent (gadolinium), the T1-weighted sequence with fat suppression is repeated in the different contrast phases in the axial and coronal planes. Magnetic resonance cholangiopancreatography was performed with T2-weighted pulse sequences.

### Statistical analysis

Data were recorded in a Microsoft Excel spreadsheet. The statistical analysis was performed with the IBM SPSS Statistics software package, version 29.0 (IBM Corp., Armonk, NY, USA). The chi-square test was used for comparing categorical variables, whereas the t-test or nonparametric tests were used for comparing continuous variables. Values of *p* < 0.05 were considered statistically significant. Multivariate logistic regression was performed to determine whether the incidental finding of PCLs (dependent variable) were associated with age, sex, hepatic cyst, and kidney cyst (independent variables). After the univariate analysis had been performed, variables significantly associated with PCLs were analyzed in the multivariate logistic regression model. Odds ratios and 95% confidence intervals were used in order to display the robustness of the association.

## RESULTS

A total of 1,296 abdominal MRI scans were performed during the study period. Of those, 1,211 met the selection criteria and were included for the analysis (Figure 1). The mean age of the patients who met the inclusion criteria was 52.55 years (range, 18–96 years), and 452 (62.68%) of those patients were male. We identified PCLs in 138 patients (11.40%). As shown in Table 1, PCLs were identified in 43 (9.51%) of the 452 male patients and in 95 (12.52%) of the 759 female patients (*p* = 0.112). Is important to note that our preliminary data were published elsewhere^(17)^.

**Table 1:** Features of patients with and without PCLs.

Feature	PCLs			*P*-value
	Total (N = 1,211)	Yes (n = 138)	No (n = 1,073)	
Sex (male/female), n/n	452/759	43/95	409/664	0.112
Mean age (years), mean ± standard deviation	52.55 ± 15.65	64.57 ± 13.15	51.01 ± 15.27	< 0.001
Liver cyst(s), n (%)	323 (26.68)	53 (38.41)	270 (25.16)	< 0.001
Kidney cyst(s), n (%)	443 (36.58)	83 (60.14)	360 (33.55)	< 0.001

**Figure 2:**
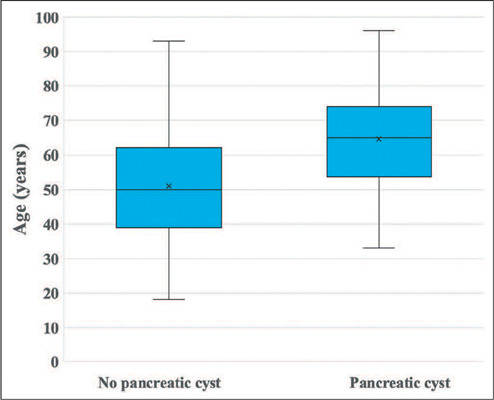
Box-and-whisker plots showing the age distribution of patients without and with incidental PCLs. The horizontal line inside each box represents the median.

**Figure 3:**
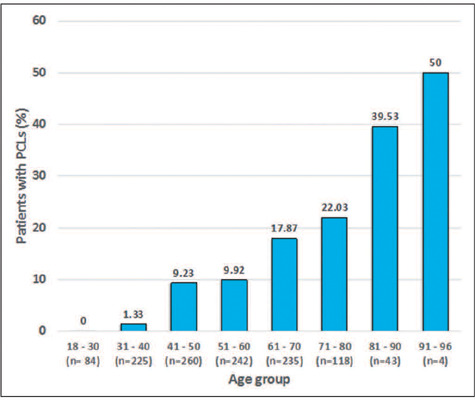
Cumulative prevalence of incidental PCLs stratified by age; that prevalence was found to increase with age.

As can be seen in Figure 2, the patients with an incidental finding of PCLs on MRI were significantly older than were those without (mean age of 64.57 ± 13.15 vs. 51.01 ± 15.27 years and median age of 65 vs. 50 years; *p* < 0.001). None of the 84 patients in the 18- to 30-year age group had PCLs. Of the 225 patients in the 31- to 40-year age group, only three (1.33%) had PCLs, compared with 24 (9.23%) of the 260 patients in the 41- to 50-year age group, 24 (9.92%) of the 242 patients in the 51- to 60-year age group, 42 (17.87%) of the 235 patients in the 61- to 70-year age group, 26 (22.03%) of the 118 patients in the 71- to 80-year age group, and 43 (39.53%) of the 43 patients in the 81- to 90-year age group. Only four patients were over 90 years of age, with the oldest being 96, and two (50%) of those four patients had PCLs. The findings by age group are summarized in Figure 3. The patients under 80 years of age had a cumulative PCL prevalence of 10.22%, compared with 40.43% for those over the age of 80 (*p* < 0.001).

Of the 138 patients with PCLs, 53 (38.41%) had liver cysts, 83 (60.14%) had kidney cysts, 35 (25.36%) had liver and kidney cysts, and 37 (26.81%) had neither. As can be seen in Table 1, the proportion of patients with liver cysts was significantly higher among those with PCLs than among those without (38.41% vs. 25.16%; *p* < 0.001), as was the proportion of patients with kidney cysts (60.14% vs. 33.55%; *p* < 0.001).

**Figure 4:**
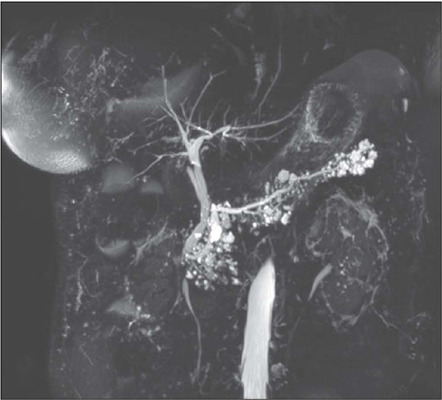
63-year-old-female diagnosed with multifocal mixed-type IPMN. Magnetic resonance cholangiopancreatography showing multiple cysts in communication with the main pancreatic duct and with well-defined homogeneous signal intensity in the pancreas. The main pancreatic duct is at a diameter of 0.6 cm in the pancreatic head.

The largest PCL had a mean diameter of 1.18 cm (range, 0.2–6.5 cm), with a median diameter of 0.95 cm (Table 2). We identified PCLs with a diameter < 2 cm in 121 patients (87.68%). Of the 138 PCLs, 32 (23.19%) were located in the head of the pancreas, one (0.72%) was in its neck (Figure 4), 34 (24.64%) were in its body, 25 (18.12%) were in its tail, and 46 (33.33%) were multifocal. The location of the cyst in the pancreas was not found to correlate with the mean diameter of the largest PCL (*p* = 0.83), the age of the patient (*p* = 0.41), or the sex of the patient (*p* = 0.34).

**Table 2:** Cyst characteristics.

Cyst characteristic	PCLs (n = 138)
Size (cm), mean (range)	1.18 (0.2–6.5)
Location in the pancreas, n (%)	
Head	32 (23.19)
Neck	1 (0.72)
Body	34 (24.64)
Tail	25 (18.12)
Multifocal	46 (33.33)
Radiological diagnosis, n (%)	
IPMN	69 (50.0)
SCN	36 (26.09)
Indeterminate	33 (23.91)

**Figure 5:**
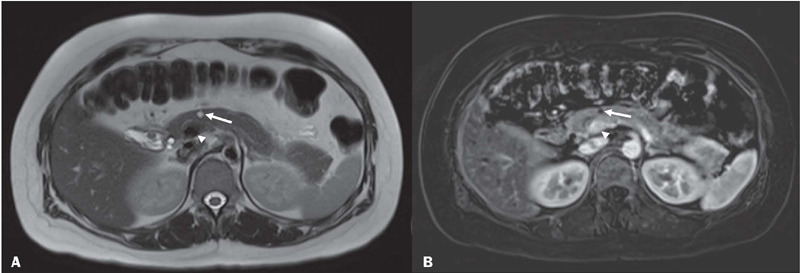
33-year-old female diagnosed with BD-IPMN without high-risk stigmata or worrisome features. **A:** T2-weighted image of the abdomen showing a 0.6 cm cystic lesion in the pancreatic neck (arrow), in communication with the main pancreatic duct. **B:** Axial image after administration of gadolinium-based intravenous contrast, showing no significant enhancement of this cyst (arrow). The arrowheads show the portomesenteric venous axis.

**Figure 6:**
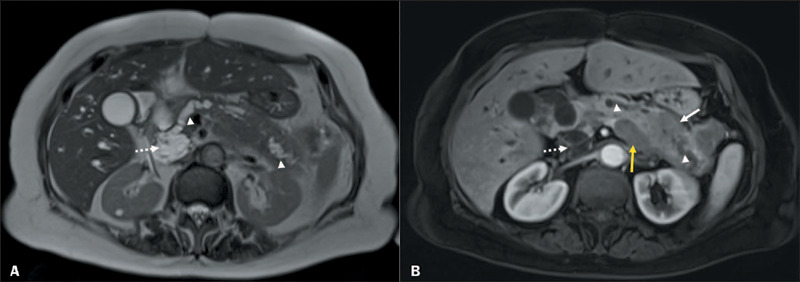
72-year-old female diagnosed with IPMN and associated invasive carcinoma. **A:** T2-weighted image of the abdomen showing a 2.9-cm cystic lesion in the head of the pancreas (dotted arrow) in communication with the main pancreatic duct (arrowheads). **B:** Image after administration of gadolinium-based intravenous contrast, showing a solid infiltrative lesion with imprecise margins (solid white arrow) in the body and tail of the pancreas, with an abrupt change in the caliber of the distal pancreatic duct (arrowheads). Note also the splenic vein thrombosis (yellow arrow).

The radiological diagnosis was IPMN in 69 patients (50.0%), SCN in 36 patients (26.09%), and indeterminate cyst in 33 patients (23.91%). Of the 69 IPMNs, 67 (97.1%) were branch duct (BD)-IPMNs, and two (2.9%) were mixed-type IPMNs. One (0.72%) of the patients with a BD-IPMN had high-risk stigmata (cystic lesion of 6.5 cm in the head of the pancreas with a solid component and obstructive jaundice) suggestive of an IPMN with associated invasive carcinoma (Figure 5). No high-risk stigmata or worrisome features were observed in any of the remaining 66 patients with BD-IPMNs or in any of those with indeterminate PCLs. The two patients with mixed-type IPMNs did not show high-risk stigmata—the diameter of the main pancreatic duct was 0.6 cm in one (Figure 6) and 0.7 cm in the other—and no other worrisome feature was reported. Of the 46 multifocal PCLs, 36 (78.26%) were IPMNs (34 BD-IPMNs and two mixed-type IPMNs), seven were indeterminate, and three were SCNs.

In the univariate analysis, age, liver cysts, and kidney cysts were associated with the incidental finding of PCLs. In the multivariate logistic regression analysis, only age and kidney cysts showed an association with the incidental finding of PCLs (Table 3).

**Table 3:** Univariate and multivariate logistic regression analyses of features associated with incidentally detected PCLs.

Feature	Univariate analysis		Multivariate analysis	
	Odds ratio (95% CI)	P	Odds ratio (95% CI)	P
Sex	1.36 (0.93–1.99)	0.112		
Age	1.35 (1.08–1.62)	< 0.001	1.06 (1.04–1.07)	< 0.001
Liver cyst(s)	1.85 (1.28–2.68)	< 0.001	1.38 (0.97–2.04)	0.114
Kidney cyst(s)	2.89 (2.08–4.29)	< 0.001	2.05 (1.35–3.09)	0.001

## DISCUSSION

In our study, the prevalence of an incidental finding of PCLs in adults undergoing MRI was 11.4%, comparable to reported in other studies of PCLs as incidental findings on MRI, although the reported prevalence varies widely across studies, ranging from 0.2% to 45.9%^(3,7,11,12,18)^. The 11.4% prevalence observed in our study and the 9.3% reported by Oliveira et al.^(12)^ indicate that the prevalence of PCLs in South America is comparable to the pooled prevalence (16%) determined from the 24%, 20%, and 5% reported for the United States, Europe, and Asia, respectively^(11)^.

The high (45.9%) prevalence reported by Kromrey et al.^(7)^ might be due to better image quality or to the fact that those authors did not exclude patients with a history of pancreatic disease or pancreatic surgery, as was done in other studies^(19,20)^.

As in the studies conducted by de Jong et al.^(19)^, Lee et al.^(3)^, and others^(7,11,20,21)^, we found that advancing age is strongly associated with an incidental finding of PCLs. In our study sample, we identified PCLs in only 0.97% of the patients under 40 years of age, which is comparable to the 0.87% reported by Lee et al.^(3)^ and the 0.78% reported by Zhu et al.^(22)^. In contrast, the prevalence of incidental PCLs among our patients over 80 years of age was 40.43%. This age-dependent difference in prevalence has led to suggestions that PCLs constitute an acquired condition, appearing and growing with age, which could explain the scarcity of PCLs in young patients^(3)^.

No correlation was found between PCLs and sex, which is in accordance with the findings of other studies^(3,7,12,19,20)^. We found that 33.33% of our patients had more than one PCL (multifocal PCLs), similar to the 40% and 44% reported by Lee et al.^(3)^ and Zhang et al.^(20)^, respectively. In our other patients, cysts were equally distributed among the sections of the pancreas, comparable to the results reported by Laffan et al.^(5)^ and de Jong et al.^(19)^.

In a recent meta-analysis, Zerboni et al.^(21)^ reported that 60% of all incidentally diagnosed PCLs are mucinous lesions (range, 35–100%). In our study, 50% of all incidental PCLs were diagnosed as IPMNs, because the cyst was in communication with the pancreatic ductal system. In addition, many PCLs are indeterminate on imaging (23.91% in our study), and the course of such PCLs therefore cannot be predicted with any certainty^(10)^.

It is well known that IPMN is one of the precursor lesions of pancreatic cancer^(16,23)^, with the capacity to progress from low-grade dysplasia to high-grade dysplasia and on to invasive carcinoma^(23)^. That pattern of progression occurs in 20–30% of cases of pancreatic cancer^(2)^. Therefore, the opportunity of a cure depends on early detection and adequate surgical resection. Identifying an IPMN at an early stage and providing adequate surveillance could prevent a precursor lesion from progressing to pancreatic cancer.

Only 0.7-5.7% of PCLs detected as incidental findings have features suspicious of malignancy and represent an indication for surgery^(7,20,21)^. In our study sample, we identified one patient (0.72%) with a BD-IPMN who also showed radiological features of associated invasive carcinoma and signs of carcinomatosis (ascites and increased peritoneal enhancement). That patient underwent computed tomography-guided biopsy confirming the diagnosis of pancreatic adenocarcinoma and is, at this writing, being treated with a chemotherapy protocol.

It is important to know and understand the real prevalence of PCLs on our continent, because it could give rise to health care policies for allocating human and technological resources for the proper approach the incidental finding of pancreatic lesions, especially in low- and middle-income countries where access to endoscopic ultrasound or MRI is limited due to their high cost and to a lack of adequate resources. Such an approach could avoid unnecessary health care expenditures and invasive procedures that could be harmful to patients.

Studies have shown that PCLs can be accompanied by cysts in other solid abdominal organs, including the liver, kidneys, and spleen^(24)^. In our study sample, liver and kidney cysts were more common among the patients with PCLs than among those without, although only kidney cysts were associated with PCLs in the multivariate analysis. We identified kidney cysts in 60.14% of our patients with PCLs. The reported prevalence of kidneys cysts in patients with PCLs ranges from 30.0% to 88.2%^(20,24,25,26)^. It is well known that most liver cysts arise from abnormalities of the ductal plate during embryonic development^(27)^, leading to cyst transformation in the epithelium of the intrahepatic bile duct, which does not communicate with the biliary tract. Kidney cysts are the result of renal ischemia, fibrosis of the medullary stroma, and fragility of the basement membrane due to age-related arteriosclerosis^(25)^. The associations that PCLs show with liver and kidney cysts cannot be explained from a histological or embryological perspective^(25)^, although our results regarding the association between PCLs and kidney cysts could be explained by age-related gene mutations. Kim et al.^(28)^ found a higher prevalence of PCLs in individuals with autosomal polycystic kidney disease with mutations in the PKD2 gene. Polycystin-2, the protein product of PKD2, could be involved in pancreatic development, when common pancreatic progenitor cells have been destined to become ductal epithelium^(28)^. In addition, it is thought that polycystin-2 is needed for the preservation of the normal pancreatic ductal system in adults^(27,29)^.

The definitive treatment of incidentally detected PCLs remains to be elucidated. However, our data offer a reference to support decisions regarding the management of PCLs and could assist in future research.

This study has some limitations. First, the observational, retrospective design could have introduced a selection bias, which we attempted to counter by including all patients who underwent abdominal MRI during the study period. In addition, the study was conducted at a single center, which could limit its generalizability. Furthermore, we were not able to establish definitive histopathological diagnoses, because MRI cannot distinguish among absent, normal, and dysplastic epithelium. Although it is currently unfeasible to improve the correlation between the radiological and histopathological diagnoses, there could be, in the future, advances in imaging techniques that will allow the PCL epithelium to be visualized.

## CONCLUSION

In our study sample, the prevalence of incidentally detected PCLs was 11.4%. That prevalence increased significantly with age, and it was not associated with sex. Half of the incidental PCLs identified were IPMNs, with a low rate of features suggesting malignant degeneration. The PCLs did not show a predilection for any particular portion of the pancreas, and a third were multifocal. We also detected an association between incidentally detected PCLs and cysts in other organs, with kidney cysts being the most strongly associated.
